# Psychological Effect of Anti-Asian Attitudes by COVID-19 on Asian Americans in Coastal Alabama

**DOI:** 10.1093/geroni/igab046.3524

**Published:** 2021-12-17

**Authors:** Hosik Min, Roma Hanks, Denise Lewis

**Affiliations:** 1 University of South Alabama, Mobile, Alabama, United States; 2 University of Georgia, Athens, Georgia, United States

## Abstract

This study aimed to understand how the anti-Asian attitude due to the COVID-19 affected Asian American communities in Alabama. We asked whether Asian Americans were worried about going out due to the anti-Asian attitude due to COVID-19. This study conducted online surveys to Cambodians or Laotians, who were 18 years and older, were living in Coastal Alabama, in May 2020. To avoid in-person contact, respondents answered questions online. A total of 353 respondents participated in the survey. In the Cambodian community, more younger adults participated in the survey, while more middle-aged adults participated from the Laotian community. Laotians had longer educational attainment and watched multiple media to obtain COVID-19 related information. Cambodians (72%) were afraid of COVID-19 infection more than Laotians (53%). More Cambodians (73%) were afraid to go out because of the anti-Asian attitude than Laotians (52%). The logistic regression analysis presented that people worried more about the COVID-19 infection were less likely to go out due to anti-Asian attitudes. Educational attainment did not have a protective effect. Watching multiple media sources decreased the worry about the anti-Asian attitude for Laotians. The age cohort showed both a protective and exacerbate the effect. Cambodians, who were in their thirties, were worried about going out. However, Laotian fifties and over did not worry about going out. This difference might be related to the length of the stay in the U.S. Hanks et al. found that Cambodians, compared to Laotians, had more new immigrants who recently came to the community to marry.

